# Sirt6 promotes tumor growth and suppresses immune surveillance

**DOI:** 10.1186/s12935-025-04125-x

**Published:** 2026-01-13

**Authors:** Yan Wang, Yu Song, Xianqin Song, Nanyang Zhang, Kehua Fang, Xiaotian Chang

**Affiliations:** 1 Medical School, Qingdao Huanghai University, Linghai road 1145, Qingdao, 266000 Shandong P. R. China; 2 Medical Research Center, The Affiliated Hospital of Qingdao University, Wutaishan road 1677, Qingdao, 266000 P. R. China; 3 Department of Pediatrics, Qilu Hospital, Shandong University, Wenhuaxilu road 107, Jinan, 250012 China; 4 Clinical Laboratory, The Affiliated Hospital of Qingdao University, Wutaishan road 1677, Qingdao, 266000 Shandong P. R. China

**Keywords:** Sirt6, UBCS039, Lao1 (IL4I1), Immune surveillance, Tumor-associated macrophage (TAM), IFN-γ, ADO, PD-1, PD-L1

## Abstract

**Background:**

Many studies have reported increased Sirt6 expression and activity in many tumor tissues, and the expression level is inversely linked to overall patient survival. This study explored how Sirt6 affects tumor growth and immune surveillance.

**Methods:**

UBCS039, a selective Sirt6 activator, was dissolved in dimethyl sulfoxide (DMSO) and intraperitoneally administered to BALB/c-nude mice. These mice were then given injections of human tumor-derived HeLa, MB-231, Lm-3, or Hcc827 cells to establish a tumor-bearing model by routine methods.

**Results:**

Compared with tumor-bearing mice pretreated with DMSO or PBS, the mice pretreated with UBCS039 showed larger tumors. The levels of granulocyte–macrophage colony-stimulating factor (GM-CSF), IL-10, adenosine (ADO) and NK cells were elevated in the peripheral blood of UBCS039-pretreated mice, and the IFN-γ level was decreased. Increased expression levels of Sirt6, PD-L1, NF-κB, and PD-1 were detected in the tumor tissues of UBCS039-pretreated mice. A greater abundance of M2 macrophages, also termed tumor-associated macrophages (TAMs), was observed in UBCS039-pretreated mouse tumors. Moreover, upregulation of novel Lao1 (interleukin 4-induced 1, IL4I1), which is known to control M2 polarization, was specifically detected in tumor tissues from UBCS039-treated mice via transcriptomic analysis and was verified by real-time PCR and western blotting. UBCS039 also stimulated M0-type and M1-type macrophage polarization to the M2-type phenotype in vitro, and Sirt6 and Lao1 expression increased during this process.

**Conclusions:**

Sirt6 can increase ADO, PD-L1 and PD-1 levels; decrease IFN-γ levels; and promote M2 polarization through the upregulation of Lao1 expression, which increases tumor growth by suppressing immune surveillance.

**Supplementary Information:**

The online version contains supplementary material available at 10.1186/s12935-025-04125-x.

## Background

 Seven class III histone deacetylases (HDACs) that are dependent on nicotinamide adenine dinucleotide (NAD+) and are extensively expressed in mammals are known as sirtuins (Sirts) [1]. Numerous tumor tissues, such as those from colon cancer, breast cancer, ovarian cancer, hepatocellular carcinoma, osteosarcoma, prostate cancer, and renal cell carcinoma, exhibit increased expression of Sirt6, a Sirt family member [2–7]. In patients with colon carcinoma, osteosarcoma or prostate cancer, Sirt6 expression level and overall survival are inversely related [5–8]. In a mouse model, MB-231 human breast cancer cell xenografts grow more slowly when Sirt6 expression is silenced [9]. The above observations suggest that Sirt6 plays an important role in tumor growth. However, how Sirt6 affects tumorigenesis remains unknown.

Some studies have discussed the involvement of Sirt6 in tumor immunity, immunometabolism, immunoregulation and immunosenescence [10–13]. For example, a study reported that Sirt6 can accelerate prostate cancer development by suppressing the innate immune response [9]. Numerous immune-related components make up the tumor immune microenvironment. The two primary types of macrophages, M1 and M2, can be polarized by many stimuli. M1 macrophages play a proinflammatory role by secreting IFN-γ. M2 macrophages are tumor-associated macrophages (TAMs) that promote tumor growth in the tumor microenvironment (TME) and control cancerous processes, such as immunological suppression, angiogenesis and tumor metastasis [14–16]. An imbalance between M1/M2 macrophages disrupts immune surveillance and promotes tumor growth [17]. Sirt6 inhibition promotes proinflammatory cytokine release by macrophages [18–20]. Additionally, adenosine (ADO) has emerged as an immune checkpoint molecule that promotes tumor escape and can impair antitumor immune responses [21]; Tumor cells’ PD-L1 attaches itself to immune cells’ PD-1 surface to start the PD-1/PD-L1 immunosuppressive program in the tumor microenvironment [22, 23]; The NF-kB cascade, which is important for immune function, is probably necessary for antitumor immunity. Reducing NF-kB activity may help slow tumor growth [24]. However, how Sirt6 affects important components of immunoregulation in the TME is not well known.

The aim of the present study was to investigate the function of Sirt6 in immune surveillance and how tumor growth is affected by it. UBCS039, a chemical, selectively activates Sirt6 and increases its degree of deacetylation. UBCS039 attaches to the acyl channel pocket unique to Sirt6 [25]. To better understand how Sirt6 functions in tumorigenesis, nude mice were treated with UBCS039 via intraperitoneal injection. Then, these mice were subcutaneously injected with human tumor cells, including HeLa cervical cancer cells, MB-231 breast cancer cells, Hcc-LM3 hepatocellular carcinoma cells, or Hcc827 non-small cell lung cancer cells to establish tumor-bearing mice. We then used positron emission tomography (PET–CT), bioluminescence in vivo imaging, and hematoxylin and eosin (H&E) staining to analyze the tissues. We also examined cytokine production, ADO levels, lymphocyte proportions, and biochemical indicators in the peripheral blood. Additionally, we used transcriptomic analysis to explore the regulatory pathways of Sirt6 in tumor tissues. Furthermore, we investigated the ratios of M1- and M2-type macrophages in tissues that received UBCS039 injections. To understand how Sirt6 affects macrophage polarization, we induced M0-, M1- and M2-type macrophages from THP-1 cells *in vitro and* treated these macrophages with UBCS039. The macrophage polarization and M1/M2 ratio were examined. We found that increased Sirt6 expression and activity caused by UBCS039 can stimulate tumor growth and increase the expression of PD-1, PD-L1 and ADO and decreased IFN-g in tumor-bearing mice. Importantly, increased Sirt6 expression and activity could elevate the expression of Lao1 (also termed IL4-induced protein 1 (IL4I1)) and induce macrophage polarization to the M2 type. These results suggest that Sirt6 promotes tumor growth and suppresses immune surveillance.

## Materials and methods

### Establishment of tumor-bearing mice and UBCS039 pretreatment

Human tumor-derived HeLa, LM-3, Hcc827, and MBA-MD-231 cells were cultured at 37 °C with 5% CO_2_ in RPMI 1640 medium (HyClone, USA) containing 1% (v/v) penicillin/streptomycin (Gibco) and 10% (v/v) FBS (Gibco). UBCS039 (MCE, China) was dissolved in dimethyl sulfoxide (DMSO; Solarbio, China) at a 20 mg/ml concentration. The EC50 of UBCS039 is 38 µM according to the product description provided by the manufacturer. Sixty five-week-old healthy female BALB/c nude mice (BALB/c FOXN1-/-) were obtained from Vital River Laboratory Animal Technology (Beijing, China). Foxn1 gene mutation leads to the development of thymus defects and T-cell deficiency in the mice. Thus, these nude mice have low resistance to infection and heterogeneous substances. They were randomly assigned to groups. Every three days, each group was given intraperitoneal UBCS039, PBS, or DMSO injections at a 10 mg/kg concentration, for a total of five injections. Each group included twenty mice. The pretreatment dosages were designed based on previous studies [26, 27]. In those studies, C57BL/6 mice were treated with UBCS039 at a concentration of 50 mg/kg.

Cultured HeLa, LM3, Hcc827 or MDA-MB-231 cells were collected and resuspended in PBS 1 day after the final UBCS039 injection into nude mice. Mice pretreated with UBCS039 received a subcutaneous injection of 1 × 10^7^ cells/ml into the right axillary area. The control mice were treated with an equal concentration of DMSO or an equal volume of PBS and injected with an equal number of cells. There were 5 mice in each subgroup. The order of treatment and measurement is random. The experiments were repeated three times.

Every animal was handled in compliance with the Helsinki Convention on Animal Protection and kept in particular conditions free of pathogens. The Ethics Committee of the Affiliated Hospital of Qingdao University licensed our study (approval number: QYFY WZLL 26622). At the end of the experiment, the mice were euthanized with an overdose (150 mg/kg) of pentobarbital sodium (Sigma‒Aldrich, China). All the experimental methods were performed in accordance with the Chinese Regulations for the Protection and Use of Animals.

### Positron emission tomography–Computed tomography (‌PET‒CT) scanning

A dose of 12 MBq of ^18^F-fluorodeoxyglucose (^18^F-FDG) was administered to UBCS039-pretreated mice through the tail vein for imaging. PET‒CT scanning was conducted using a MadicLab PSA094 PET‒CT apparatus (Madic Technology, China). Madic Technology’s Pmod software (MadicLab) was used to perform PET‒CT image fusion. The following formula was used to determine the standardized uptake value (SUV) of the tumor tissues: SUV = region of interest activity concentration (MBq/ml)/injection activity concentration (MBq/ml)/body weight (kg).

### Bioluminescence imaging

Luciferase-positive MB-231 cells were generated using a lentiviral vector system (GeneChem, China). Luciferase-positive cells (1 × 10^7^/ml) in PBS were injected subcutaneously into UBCS039-pretreated mice as described above. Bioluminescence images of tumor growth were obtained using a NightOWL LB 983 instrument (Berthold Technologies, Germany) after intraperitoneal injection of 150 mg/kg d-luciferin.

### Histopathological observation

Tumor tissues were collected and preserved in 4% paraformaldehyde (PFA). The tissues were subjected to routine ethanol-induced dehydration, paraffin embedding, microtome sectioning, and H&E staining. Physicians in our pathology department examined histopathological alterations under a light microscope.

### Quantifying cytokine levels in mouse serum

Blood samples were collected from experimental mice and centrifuged to obtain the serum. A mouse inflammatory factor panel (13-plex) (BioLegend, USA) and an Apogee A50 flow cytometer (NovoCyte D2040R, UK) were used to measure cytokine levels in the serum samples. BioLegend’s Legend_plex_v8.0 software (USA) was used to analyze the serum concentrations of granulocyte–macrophage colony-stimulating factor (GM-CSF), interferon-β (IFN-β), interferon-γ (IFN-γ), interleukin-1α (IL-1α), interleukin (IL-1β), interleukin-6 (IL-6), interleukin-10 (IL-10), interleukin-12p70 (IL-12p70), interleukin-17 A (IL-17 A), interleukin-23 (IL-23), interleukin-27 (IL-27), monocyte chemoattractant protein-1 (MCP-1) and tumor necrosis factor-α (TNF-α).

### Biochemical examination of peripheral blood

Peripheral blood samples from experimental animals were collected into tubes containing heparin. Mindray’s automatic biochemical analyzer (China) was used to measure aspartate aminotransferase (AST), alanine aminotransferase (ALT), calcium (Ca), total protein (TP), triglyceride (TG), alkaline phosphatase (ALP), uric acid (UA), creatinine (CREA), lactate dehydrogenase (LDH), urea (UREA), glucose (GLU), and phosphorus (P) plasma concentrations.

### Detecting lymphocyte subtypes in peripheral blood

Peripheral blood samples from experimental animals were collected into tubes containing ethylene diamine tetra acetic acid. The samples were incubated with a series of flow cytometry antibodies. We used eBioscience’s PE-conjugated anti-mouse CD19, APC-conjugated anti-mouse CD3e, and Percp-conjugated anti-mouse CD45 antibodies (USA) to identify CD45 + CD3-CD19 + B cells; we used eBioscience’s APC-conjugated anti-mouse CD3e, PE-conjugated anti-mouse NK1.1 and Percp-conjugated anti-mouse CD45 antibodies (USA) to detect CD45 + CD3e- NK cells. The proportions of B cells and NK cells were measured by calculating their proportions with respect to the total number of CD45 + immune cells, although there were no T cells in the nude mice.

### ADO assay

Abcam’s assay kit for fluorometric ADO (Cambridge, USA) was used to measure ADO levels in the peripheral blood of the mice. A Swiss Infinite M Plex microplate reader (TECAN, Switzerland) was used to measure the fluorescence intensity.

### Real-time quantitative polymerase chain reaction (real-time PCR)

Takara’s RNAiso Plus (Japan) was used to extract total RNA from the tumor tissues of the tumor-bearing mice. The isolated RNA was reverse-transcribed into complementary deoxyribonucleic acid (cDNA) using Vazyme’s HiScript III RT SuperMix (China). After the cDNA was added to Vazyme’s ChamQ Universal SYBR qPCR Master Mix, the target genes’ mRNA expression was assessed through fluorescence-based real-time quantitative PCR using a LightCycle96 device (Roche, Switzerland). The 2^−ΔΔ^ CT method was used to measure relative mRNA expression. The internal control was β-actin mRNA. The primer sequences are listed in Supplementary File 1.

### Immunofluorescent assay

Nude mouse tumor tissues were embedded in Sigma‒Aldrich’s 5% low-gelling-temperature agarose (type VII-A), fixed overnight at 4 °C in a periodate-lysine‒PEA solution, and sectioned using a microtome. The tissue sections were incubated with Servicebio’s (China) Cy5-labeled anti-mouse CD163 antibody, SpGreen-labeled anti-mouse CD86 antibody, and Sporang-labeled anti-mouse F4/80 antibody (China). Green (the CD86 marker) indicated M1-type macrophages, pink (the CD163 marker) indicated M2-type macrophages, and red (the F4/80 marker) indicated mature macrophages. After washing with PBS, the sections were stained with 4’,6-diamidino-2-phenylindole (DAPI). 3DHISTECH’s PANNORAMIC panoramic tissue section scanner (Hungary) helped us capture the images. 3DHISTECH’s CaseViewer 2.4 software (Hungary) was utilized to examine the images. Halo v3.0.311.314 module INDICALABS-HIPPLEX FL V3.1.0 (Indica labs, USA) was used to measure the number of positive cells as well as the total number of cells in each tissue section.

### Transcriptomic analysis of tumor tissues from tumor-bearing mice

In tumor-bearing mice that were pretreated with UBCS039, total RNA was extracted from tumor tissues, which was then reverse-transcribed into cDNA. cDNA sequencing was completed using an Illumina platform. The reads’ sequences were aligned with the designated reference genome using HISAT2 (Hical Indexing for Spliced Alignment of Transcripts; Johns Hopkins University, USA). The number of protein-coding genes in each sample was determined by sequence similarity comparison. The fold change (FC) and level of gene expression were estimated using the base mean value. FC > 1.5 and *P* < 0.05 were used to identify differentially expressed genes (DEGs). The enriched pathways were determined based on the top DEGs using the Kyoto Encyclopedia of Genes and Genomes (KEGG). Each entry’s -log10 p value was used to sort the results, which were then presented from highest to lowest values. The controls were expression profiles from tumor-bearing mice that had been pretreated with either DMSO or PBS and then injected with tumor cells.

### Western blot analysis

Total protein was extracted from tumor tissues from tumor-bearing mice that had received UBCS039 pretreatment. After the protein samples were separated using 12% SDS–PAGE, the proteins were transferred to Merck Millipore PVDF membranes (USA). Rabbit monoclonal antibodies against mouse Lao1 were purchased from Abcam (USA). GAPDH expression was used as an internal control and was detected with an anti-GAPDH antibody (Elabscience, China). NIH ImageJ software (USA) was used to measure the relative protein expression levels.

A similar protocol was used to examine Lao1 expression in cultured macrophages. Rabbit monoclonal antibodies against human Lao1 were also obtained from Abcam.

### Macrophage culture and polarization

Human monocytic THP-1 cells were commercially provided by Wuhan’s Procell Life Company (China). The cells were cultivated in high-glucose Dulbecco’s modified Eagle medium (DMEM, Procell), which was supplemented with 10% fetal bovine serum (FBS, Procell). THP-1 monocytes were cultured for 24 h in DMEM (without serum) containing 320 nM phorbol 12-myristate 13-acetate (PMA, Sigma‒Aldrich) to differentiate into M0-type macrophages. M1 macrophages were induced from M0 macrophages in DMEM (without serum) supplemented with 20 ng/mL IFN-γ (MCE, China) and 10 ng/mL LPS (Sigma‒Aldrich). M2 macrophages were induced from M0 macrophages in DMEM (without serum) supplemented with 20 ng/mL interleukin 4 (IL-4) and 20 ng/mL interleukin 13 (IL-13) (MCE, USA). The three subtypes of macrophages were rinsed with PBS and cultivated for 24 h in fresh DMEM (without serum). Genin et al. described the induction process [28].

Total RNA was isolated from macrophages and then reverse-transcribed into cDNA. Macrophage subtypes were identified via real-time PCR with primers against human CD206, Arg, TNF-α and iNOS (Supplementary File 1). Macrophage subtypes were also identified using flow cytometry.

### Macrophage treatment with UBCS039

THP-1 cells were seeded on a six-well plate (1 × 10^6^ cells/well). The cells were then stimulated to differentiate into the three macrophage subtypes as described above. Following the completion of polarization, the macrophages were thoroughly washed with PBS. UBCS039 at concentrations ranging from 75 µM was added to the wells containing each type of macrophage. The concentration was determined based on our previous study [29, 30]. Others used UBCS039 at a concentration of 100 µM to culture cells in vitro [31, 32]. Real-time PCR was used to determine Lao1 expression, and flow cytometry and real-time PCR were used to identify the different subtypes.

### Macrophage subtyping using flow cytometry

Cultured macrophages were exposed to either UBCS039 or DMSO. BioLegend APC-conjugated anti-human CD86 antibody and FITC-conjugated anti-human CD68 antibody were used to identify M1-type macrophages (CD68 + CD86+). BioLegend’s anti-CD206-PE antibody and anti-CD68-FITC antibody were used to identify M2-type macrophages (CD68 + CD206+). The samples were incubated with anti-CD206, anti-CD86 and anti-CD68 antibodies for 90 min at 4 °C in the dark. The cell types were determined using a NovoCyte D2040R ACEA flow cytometer (USA). The M1/M2 ratio was calculated by dividing the M1-type percentage by the M2-type percentage.

### Statistical analysis

The tumor growth data, blood biochemistry data, cytokine level data, immunostaining density semiquantification data, western blotting semiquantification data and flow cytometry data were analyzed via GraphPAD Prism 8.0. If the data were normally distributed, one-way ANOVA was used to analyze the significant differences among multiple groups, and the LSD-T test was used for comparisons between two groups. If the data did not fit a normal distribution or homogeneity of variance, the Kruskal‒Wallis test was applied to analyze significant differences among multiple groups. Differences were deemed statistically significant when *P* < 0.05.

## Results

### UBCS039 treatment increases tumor growth in tumor-bearing mice

**Fig. 1:**
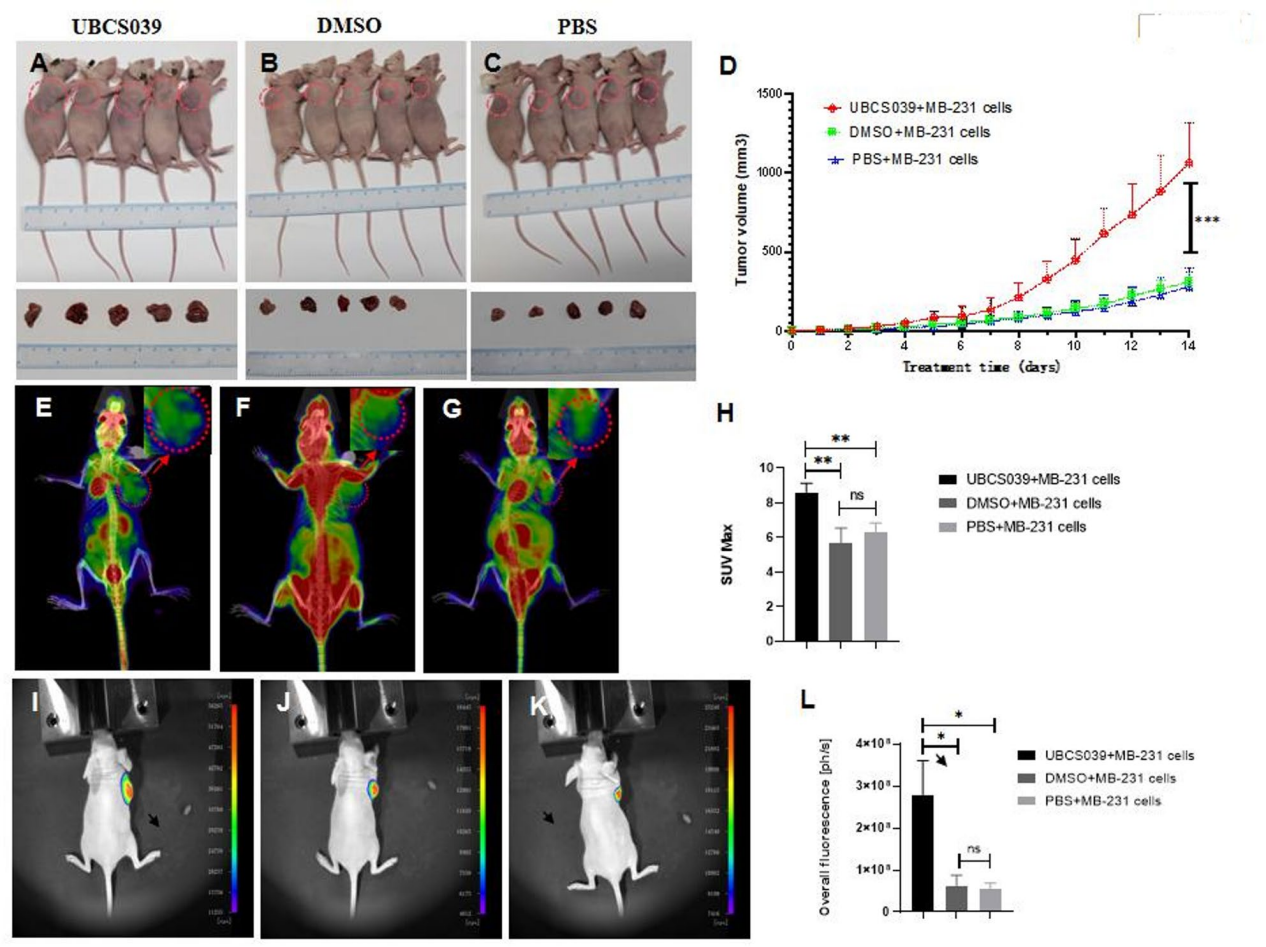
Tumor growth of nude mice subcutaneously injected with MB-231 cells following UBCS039 treatment. The mice were intraperitoneally injected with UBCS039 (**A)**, DMSO **(B)** or PBS (**C)**. (**D)** Tumor volumes were compared. Representative PET‒CT images of the mice pretreated with UBCS039 (**E)**, DMSO (**F)** or PBS (**G)**. The red dotted-line circles indicate tumors. Each group included 5 mice. The SUV values of the tumor region were compared (**H**). The representative images of bioluminescence examination for the mice pretreated with UBCS039 (**I**), DMSO (**J**) or PBS (**K**). Each group included 5 mice. The fluorescence intensities were compared (**L**). The mice pretreated with UBCS039 presented significant increases in tumor growth and metabolism. The least significant difference (LSD) test was used for comparisons between two groups. * indicates *P* < 0.05, ** indicates *P* < 0.01, *** indicates *P* < 0.001

BALB/c nude mice were given intraperitoneal UBCS039, DMSO or PBS injections. Pretreated mice were then injected with MB-231 cells. Eight days later, all UBCS039-treated mice had larger tumors than the DMSO- or PBS-treated control mice did, and this difference in tumor volume gradually increased until the mice were sacrificed on the 14th day after cell injection. No obvious differences in tumor volume were observed between the DMSO-injected mice and the PBS-injected control mice (Fig. 1A-D). PET‒CT revealed a distinct area with strong metabolic activity in the tumors of the nude mice. Furthermore, mice that were given UBCS039 presented greater SUVs in their tumors than did mice that were given DMSO or PBS (Fig. 1E-H). Additionally, HE staining revealed typical pathological features of the tumor tissues, including diffuse lamellar growth, obvious anisotropy, a spindle shape with high levels of karyopyknosis, a large and hyperchromatic nucleus, scarce cytoplasm and mitosis in many cells of the tumor tissues. There was no difference in tumor tissue pathology among UBSCS039-, DMSO- or PBS-pretreated mice (Supplementary file 2). We also injected fluorescent MB-231 cells into a different nude mouse set. Compared with control mice, UBCS039-treated mice presented more pronounced bioluminescent signals in their tumors when given either PBS or DMSO, indicating that the tumors in UBCS039-treated mice grew faster and larger than those in control mice (Fig. 1I-L). The above imaging and pathological examinations confirmed that UBCS039 pretreatment increased tumor growth in the nude mice.

**Fig. 2:**
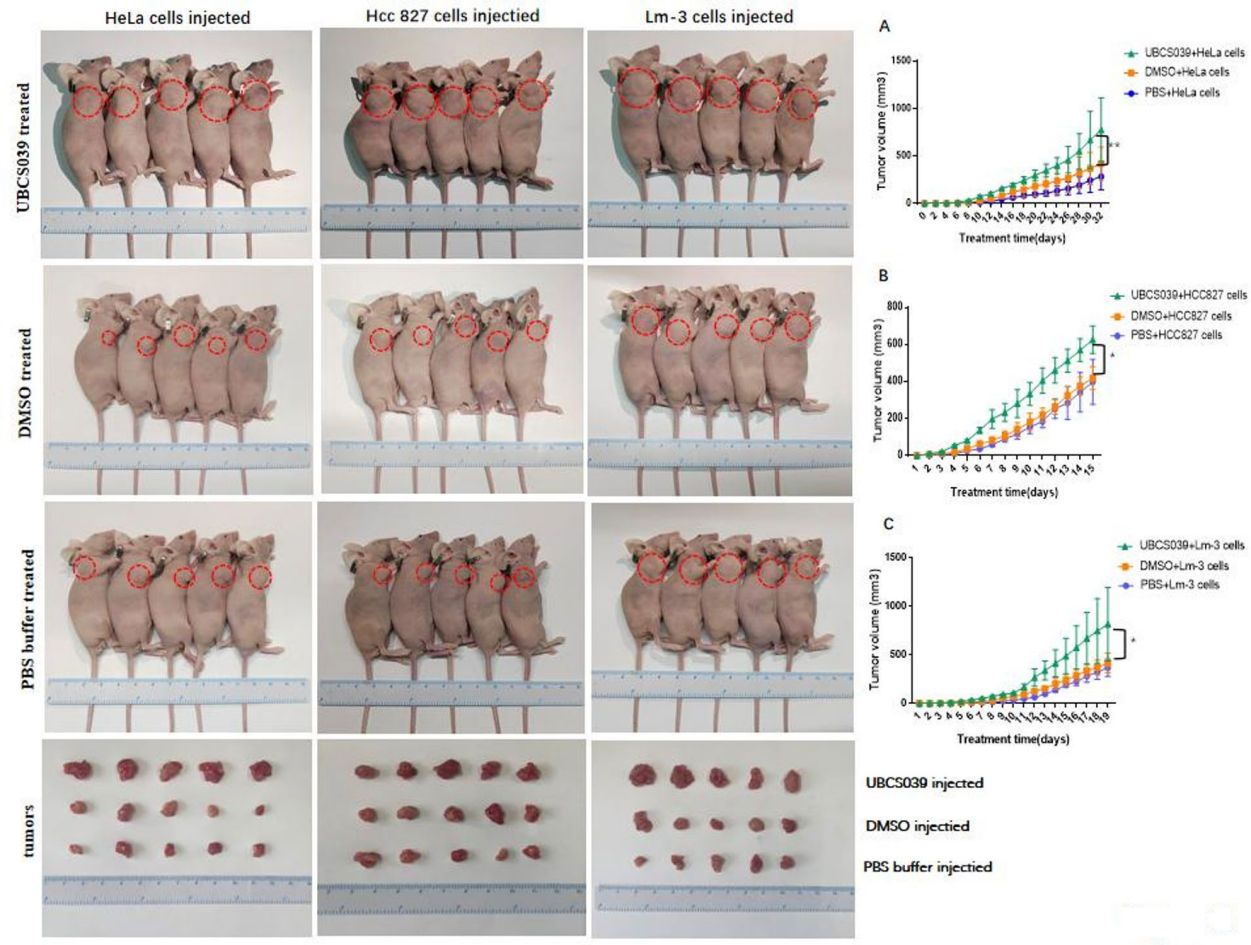
Tumor growth of nude mice subcutaneously injected with HeLa, Hcc827 or Lm-3 cells following UBCS039 treatment. The growth of tumors derived from HeLa cells (**A)**, Hcc827 cells (**B)** or Lm-3 cells (**C)** was compared. The red dotted-line circles indicate tumors. The mice pretreated with UBCS039 presented a significant increase in tumor volume. The least significant difference (LSD) test was used for comparisons between two groups. * indicates *P* < 0.05, and ** indicates *P* < 0.01

We verified the above observations by intraperitoneally injecting BALB/c nude mice with UBCS039, followed by HeLa, HCC827 or Lm-3 cell injection. Compared with control DMSO- and PBS-treated mice, all UBCS039-treated mice grew larger tumors derived from HeLa, HCC827 or Lm-3 cells, and the difference in tumor volume gradually increased until 15, 19 or 32 days after cell injection. (Fig. 2). The above experiments confirmed that UBCS039 treatment promotes the growth of various tumors. We measured the body weight of tumor-bearing mice. The tumor-bearing mouse weight began to decrease in the middle of the experiment. Compared with the tumor-bearing mice injected with PBS and DMSO, the tumor-bearing mice injected with UBCS039 lost the most weight. The weight loss of the first two groups was essentially equivalent (Supplementary file 3).

### UBCS039 treatment affects immune surveillance in tumor-bearing mice

**Fig. 3:**
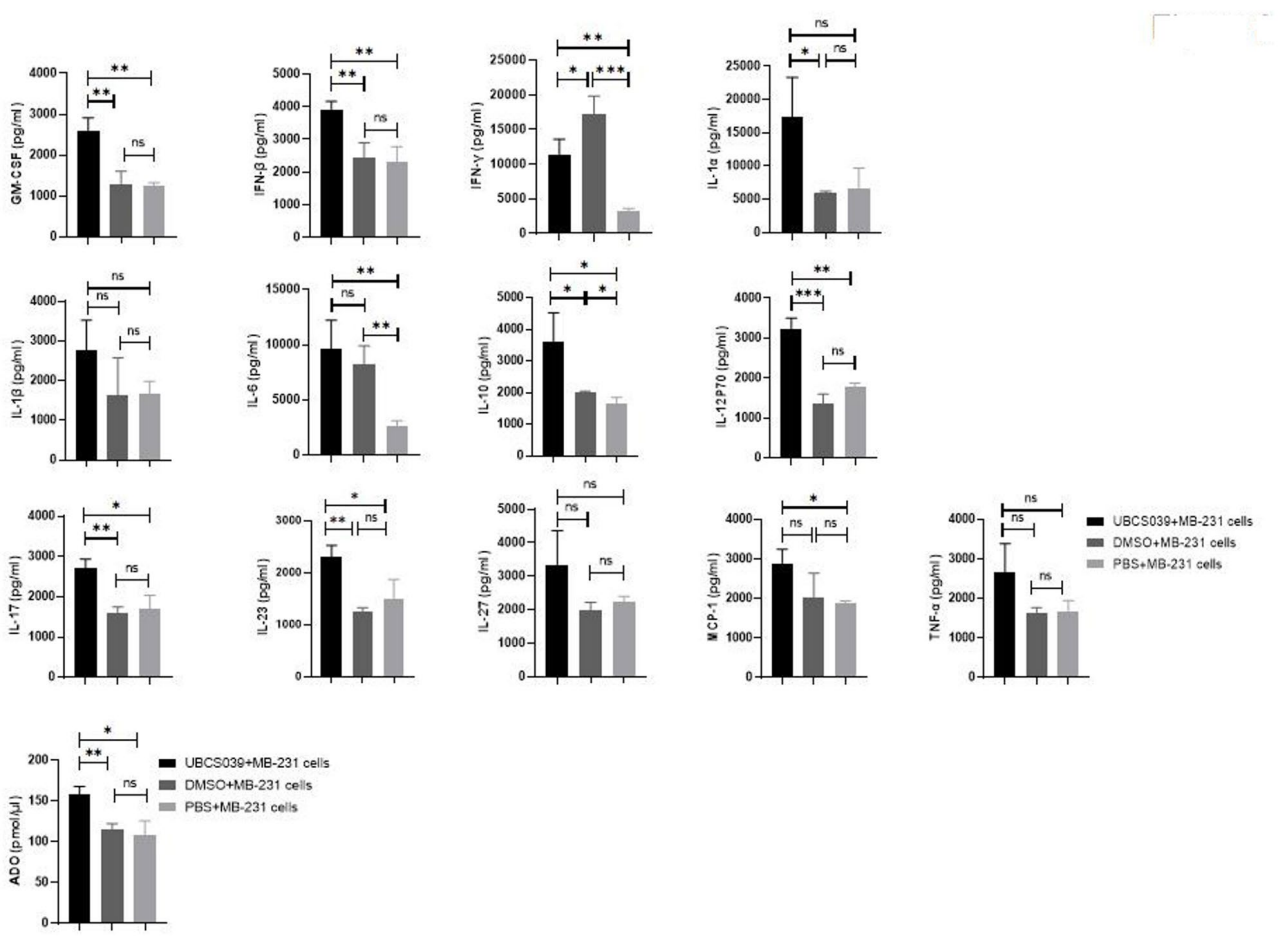
Cytokine and ADO levels in the peripheral blood of tumor-bearing mice subcutaneously injected with MB-231 cells. Cytokine levels were determined via flow cytometry, and ADO levels were determined via ELISA. The levels of ADO, GM-CSF, IFN-γ, IL-1α, IL-10, IL-12p70, IL-17 and IL-23 in the peripheral blood of tumor-bearing mice pretreated with UBCS039 significantly changed. The least significant difference (LSD) test was used for comparisons between two groups. * indicates *P* < 0.05, ** indicates *P* < 0.01, ** indicates *P* < 0.01, and *** indicates *P* < 0.001

**Fig. 4:**
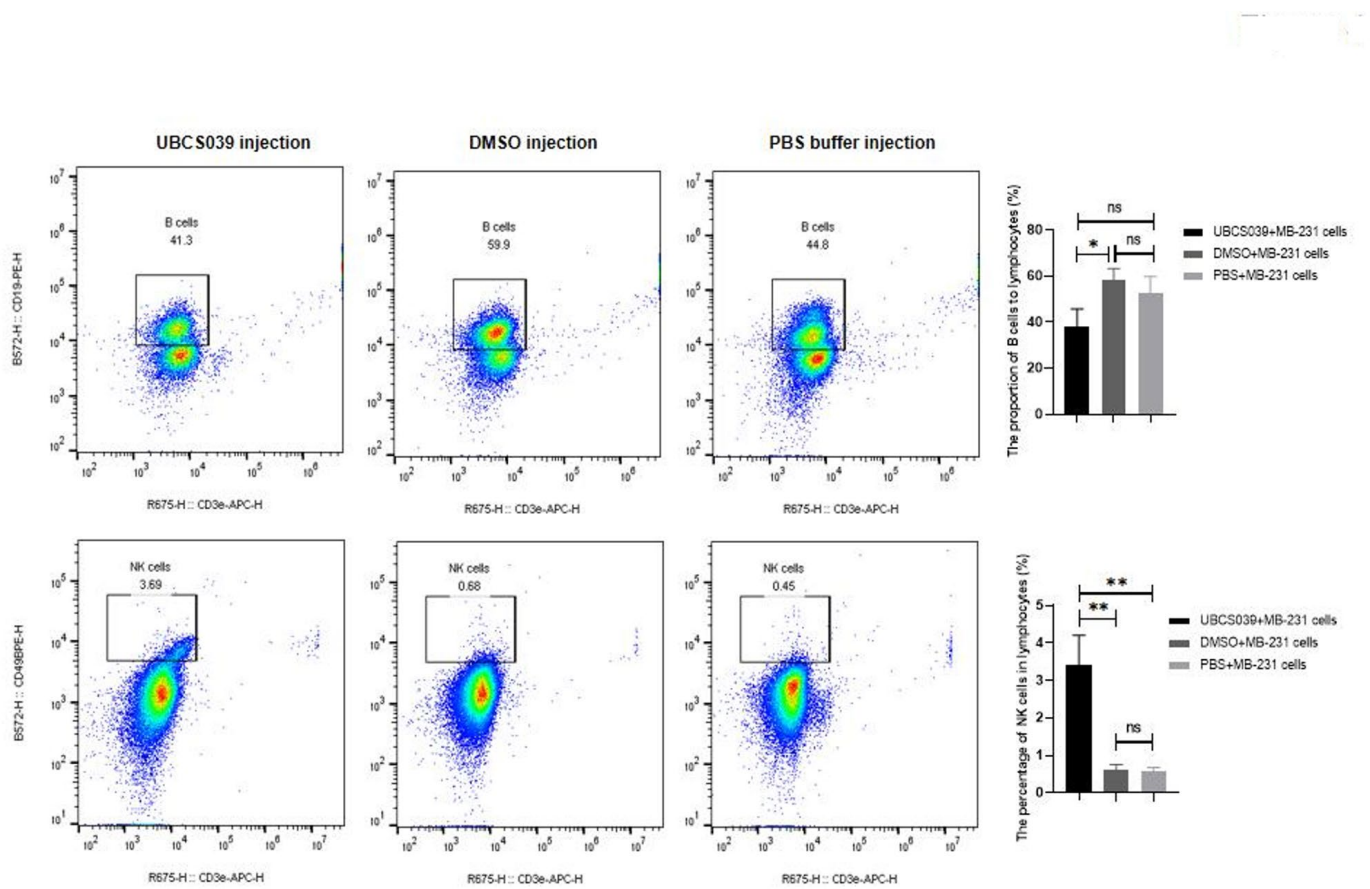
B and NK cell proportions and representative flow cytometry plots of the peripheral blood of tumor-bearing mice subcutaneously injected with MB-231 cells. The proportion of NK cells among total lymphocytes was elevated in the tumor-bearing mice following UBCS039 treatment. The least significant difference (LSD) test was used for comparisons between two groups. * indicates *P* < 0.05, and ** indicates *P* < 0.01

Compared with PBS-pretreated mice and DMSO-pretreated tumor-bearing mice, UBCS039-pretreated mice presented increased C-reactive protein (CRP) levels. Other blood biochemical indexes showed no statistically significant changes (Supplementary File 4). Moreover, GM-CSF, IFN-β, IL-10, IL-17, IL-12p70 and IL-23 levels in the serum were elevated in tumor-bearing mice that were given UBSC039; nonetheless, the IFN-γ level decreased (Fig. 3). Furthermore, peripheral blood ADO levels were significantly increased in mice that were pretreated with UBCS039 (Fig. 3). The proportion of NK cells among total lymphocytes was significantly greater than 4-fold greater in UBCS039-pretreated mice than in control mice. UBCS039 treatment did not affect the B-cell proportion in mice with tumors (Fig. 4). The results indicated that UBCS039 administration significantly altered immunomodulation-related cytokine levels and cells in these mice.

**Fig. 5:**
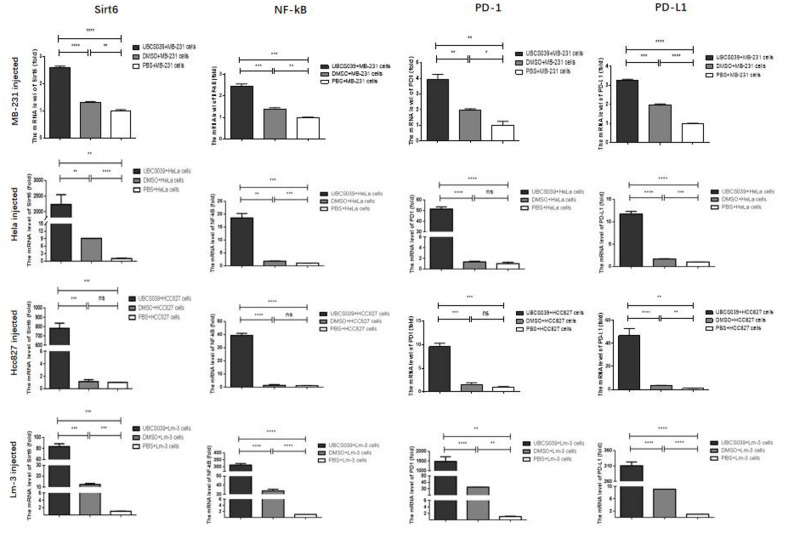
Expression levels of Sirt6, NF-κB, PD-1 and PD-L1 in the tumor tissues of tumor-bearing mice. These nude mice were subcutaneously injected with MB-231, HeLa, HCC827 or Lm-3 cells following UBCS039 treatment. The expression levels were determined via real-time PCR. The mRNA levels of Sirt6, NF-B, PD-1 and PD-L1 in the tumor tissues were significantly greater in the nude mice pretreated with UBCS039. The least significant difference (LSD) test was used for comparisons between two groups. * indicates *P* < 0.05, ** indicates *P* < 0.01, *** indicates *P* < 0.001, and **** indicates *P* < 0.0001

Real-time PCR was carried out to determine the mRNA expression of Sirt6 and other genes involved in immunoregulation in tumor tissues from MB-231, HeLa, HCC827 or Lm-3 cell-injected nude mice. Mice that received UBCS039 presented significantly greater expression of the Sirt6, NF-kB, PD-1 and PD-L1 mRNAs in tumor tissue than did mice that received either DMSO or PBS pretreatment (Fig. 5). The above results indicate that in tumor tissues, UBCS039 treatment increased Sirt6, PD-L1, NF-kB and PD-1 expression, indicating alterations in immune regulation.

**Fig. 6:**
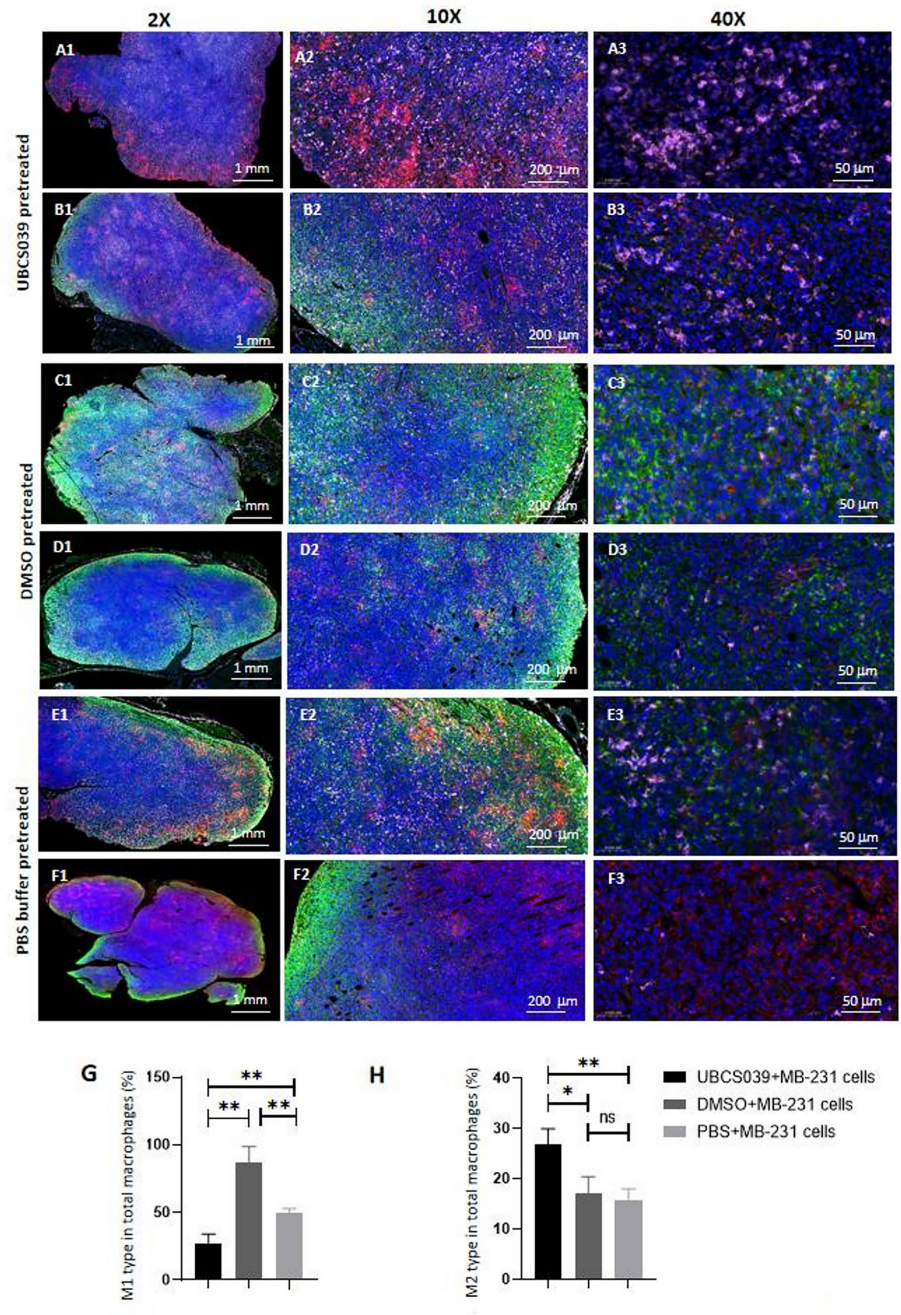
Immunofluorescent locations of macrophages in the tumor tissues of tumor-bearing mice subcutaneously injected with MB-231 cells. Tumor tissues were collected from the mice pretreated with UBCS039 (**A**,** B**) or the mice pretreated with DMSO (**C**,** D**) or PBS (**E**,** F**). Red staining with an anti-F4/80 antibody indicates mature macrophages, green staining with an anti-CD86 antibody indicates M1 macrophages, and pink staining with an anti-CD163 antibody indicates M2 macrophages. The proportions of M1 macrophages among total macrophages (**G**) and of M2 macrophages among total macrophages (**H**) were compared. Immunofluorescent signals of M2 macrophages were significantly increased in the tumor tissues of UBCS039-pretreated mice. The least significant difference (LSD) test was used for comparisons between two groups. * indicates *P* < 0.05, and ** indicates *P* < 0.01

Macrophage distribution in MB-231 cell-derived tumor tissues from UBCS039-pretreated nude mice was examined by immunofluorescent assay. We used CD86 and CD163 as immunostains for M1 and M2 macrophages, respectively, and total mature macrophages were immunostained for F4/80. They are shown in green, pink and red, respectively, and the proportions of the three macrophage types were calculated on the basis of their signal densities relative to the total immunosignals of mature macrophages in the tissue sections. M1-like macrophages with green labeling were located mainly at the tumor edge and were relatively abundant in tumor tissues from DMSO-pretreated mice and PBS-pretreated mice. However, M1-like macrophages were not abundant in tumor tissues from UBS039-pretreated mice. Pink-labeled M2-like macrophages were found at the internal as well as tumor tissue margins, and a relatively high number of these macrophages were found in the tumor tissues of the mice that had received UBCS039 pretreatment. Semiquantitative analysis of the immune signal density revealed a high percentage of M2-type macrophages among the total macrophages in tumor tissues from UBCS039-pretreated mice (Fig. 6). Real-time PCR also revealed that CD206 and Arg1, the gene markers of M2 macrophages, were more highly expressed in the tumor tissues from MB-231, HeLa, HCC827 and Lm-3 cell-injected mice following UBCS039 treatment, whereas TNF-α and iNOS, the gene markers of M1 macrophages, were more highly expressed in these tissues from the mice subjected to DMSO or PBS pretreatment (Supplementary file 5). Both measurements revealed that the number of M2 macrophages was greater in the tumors of the mice that were given UBCS039 than in the tumors of the control mice, suggesting that UBCS039 treatment increased the number of M2 macrophages.

### UBCS039 specifically increased Lao1 (IL4I1) expression in the tissues

**Fig. 7:**
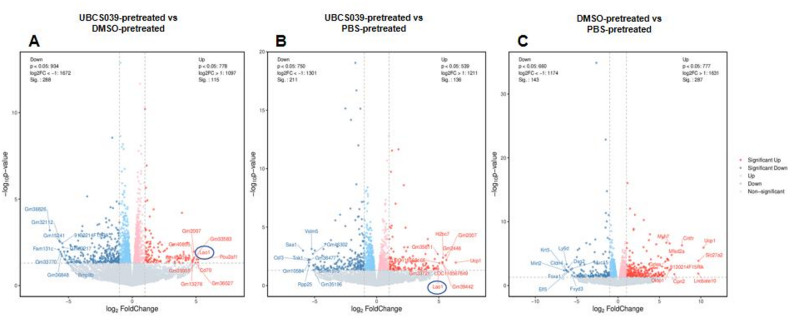
The expression profiles of tumor tissues from MB-231 cell-injected mice. Tumor tissues were collected from the tumor-bearing mice and subjected to transcriptomic analysis. The top 20 DEGs with upregulated (red) and downregulated (blue) gene expression are shown. (**A)** Expression profiles of tumor tissues from UBCS039-treated mice were compared with those from DMSO-pretreated mice. (**B)** Expression profiles of UBCS039-pretreated mice were compared with those of PBS-treated mice. **(C)** The expression profiles of DMSO-pretreated mice were compared with those of PBS buffer-pretreated mice. UBS039 treatment specifically elevated Lao1 expression (blue cycle) in tumor tissues

**Fig. 8:**
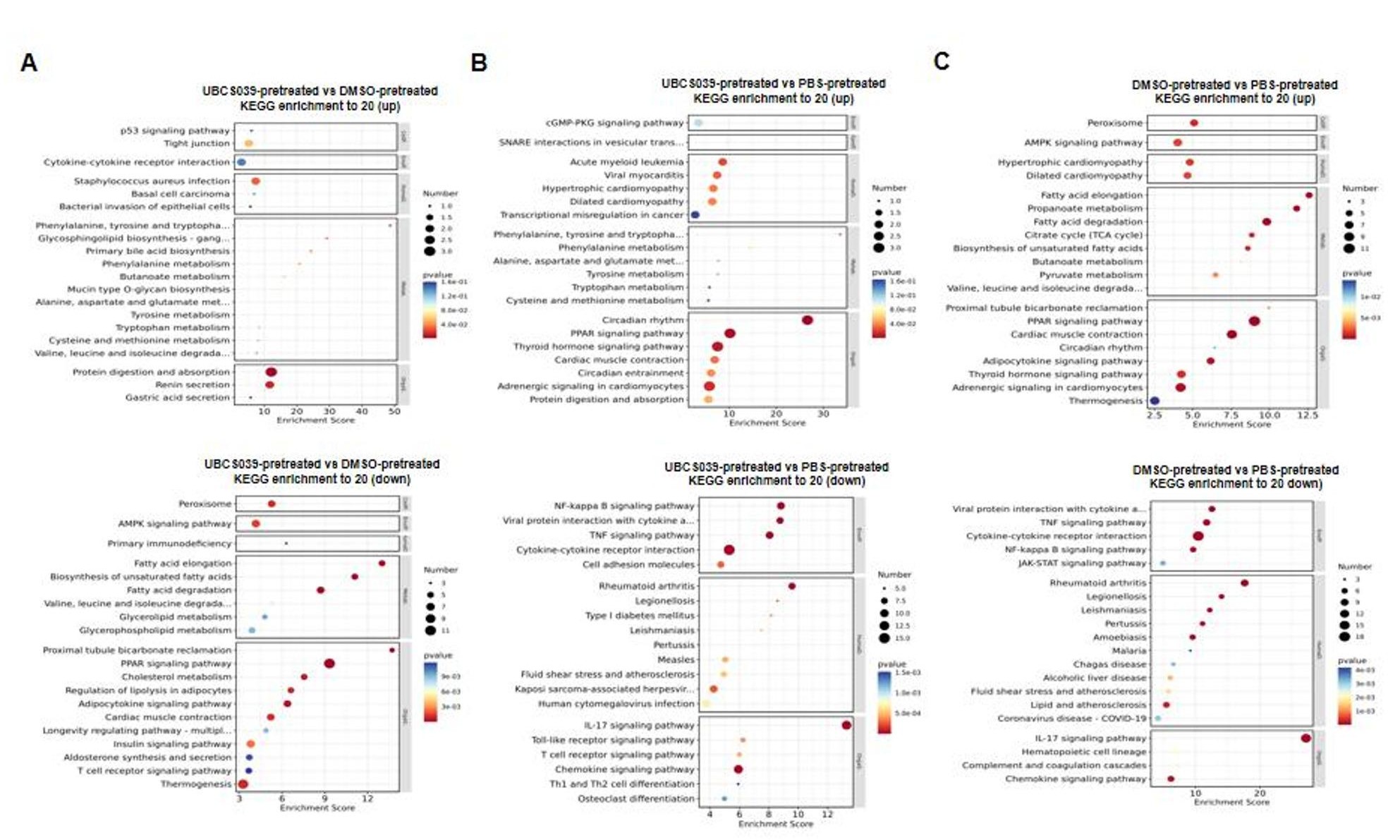
KEGG analysis of the expression profiles of tumor tissues from MB-231 cell-injected mice. (**A)** Expression profiles of tumor tissues from UBCS039-pretreated mice were compared with those from DMSO-pretreated mice. (**B)** Expression profiles of tumor tissues from UBCS039-pretreated mice were compared with those from PBS-injected mice. (**C)** Expression profiles of tumor tissues from DMSO-pretreated mice were compared with those from PBS-injected mice

Tumor tissues from MB-231 cell-injected nude mice were examined via transcriptomic analysis. Tumor tissues from UBCS039-pretreated mice presented different expression profiles than those from DMSO-pretreated mice and PBS-pretreated mice. Lao1 (L-amino acid oxidase, interleukin 4-induced 1, IL4I1) was the only common DEG according to the comparisons of tumor expression profiles between mice that were given UBCS039 or DMSO and between mice that were given UBCS039 or PBS. Lao1 expression did not significantly differ between the tumors of the mice that were given DMSO and those of the mice that were given PBS (Fig. 7), indicating that Lao1 expression was particularly increased by UBCS039 treatment. On the basis of the DEGs discovered through examination of the tumor expression profiles, we carried out a KEGG enrichment study. Comparisons of the expression profiles between UBCS039-pretreated mice and DMSO-pretreated mice, and between UBCS039-pretreated mice and PBS-pretreated mice, revealed enrichment in tyrosine and tryptophan biogenesis, tyrosine metabolism, phenylalanine, phenylalanine metabolism, aspartate and glutamate metabolism, alanine, cysteine and methionine metabolism, protein digestion and absorption, and tryptophan metabolism, while a decrease in the T-cell receptor signaling pathway was commonly observed. KEGG analysis did not detect commonly enriched pathways by comparing tumor expression profiles between mice that were given DMSO or PBS, indicating that UBCS039 specifically modulates these mentioned pathways (Fig. 8).

**Fig. 9:**
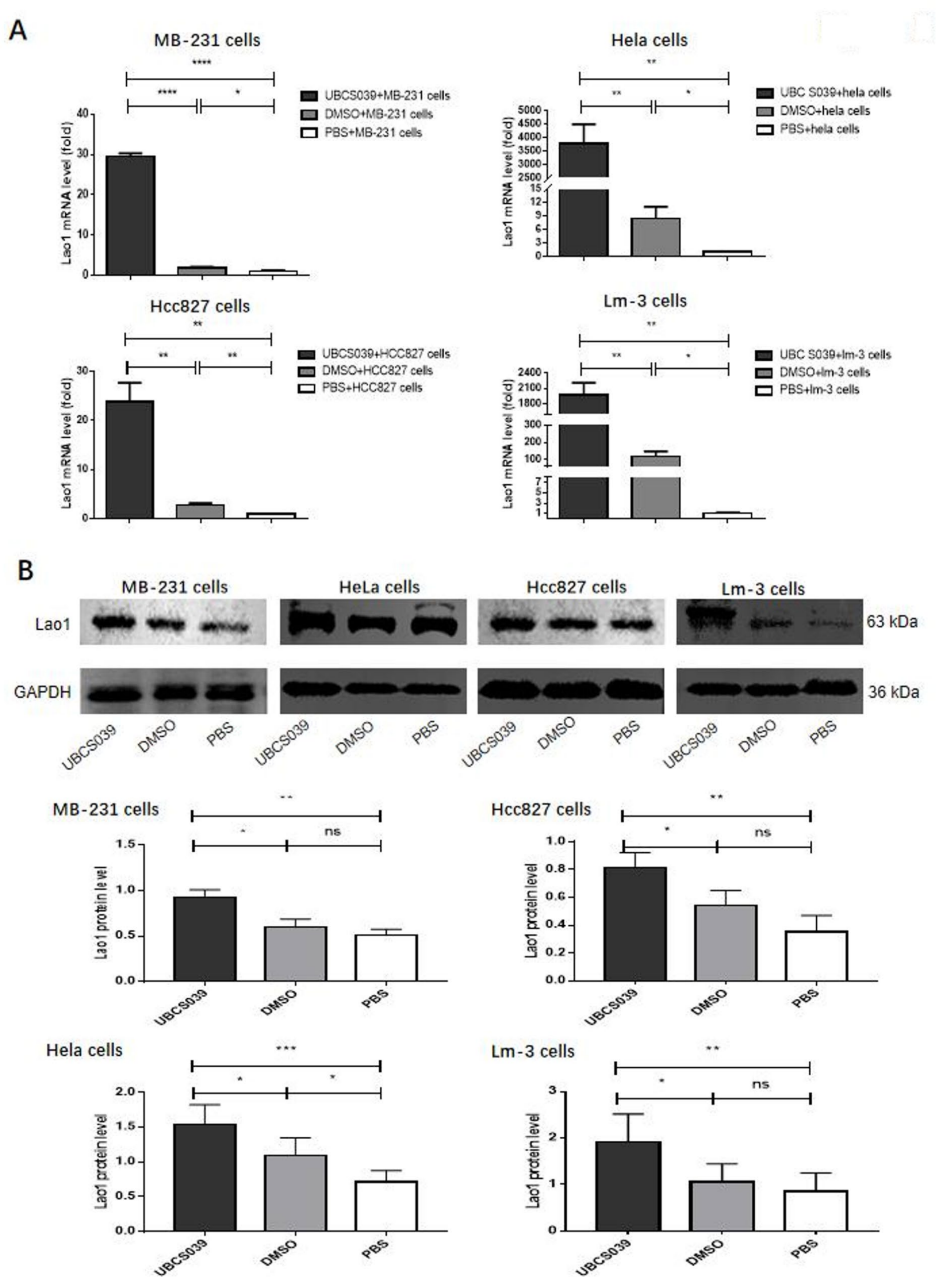
Lao1 expression in the tumor tissues of nude mice subcutaneously injected with MB-231, HeLa, HCC827 or Lm-3 cells following UBCS039 treatment. (**A)** Real-time PCR revealed increased expression of Lao1 in the tumor tissues of nude mice pretreated with UBCS039. (**B**) western blot analysis confirmed the increased expression of Lao1 after normalization of its expression to that of GAPDH. The least significant difference (LSD) test was used for comparisons between two groups. * indicates *P* < 0.05, ** indicates *P* < 0.01, *** indicates *P* < 0.001, and **** indicates *P* < 0.0001

Real-time PCR was used to measure Lao1 mRNA expression in tumors from MB-231, HeLa, HCC827 or Lm-3 cell-injected nude mice. Compared with mice that were given either DMSO or PBS, mice that were given UBCS039 presented significantly increased Lao1 mRNA expression in tumor tissues (Fig. 9A). The increase in Lao1 expression in tumor tissues from UBCS039-treated mice was also verified by western blotting (Fig. 9B). This process was repeated 3 times. The whole western blot membrane and the original data are shown in Supplementary file 6. These results indicate that the upregulation of Sirt6 by UBCS039 increased Lao1 expression in tumor tissues.

### UBCS039 promoted macrophage polarization to the M2-type and elevated Lao1 and Sirt6 expression during this process

**Fig. 10:**
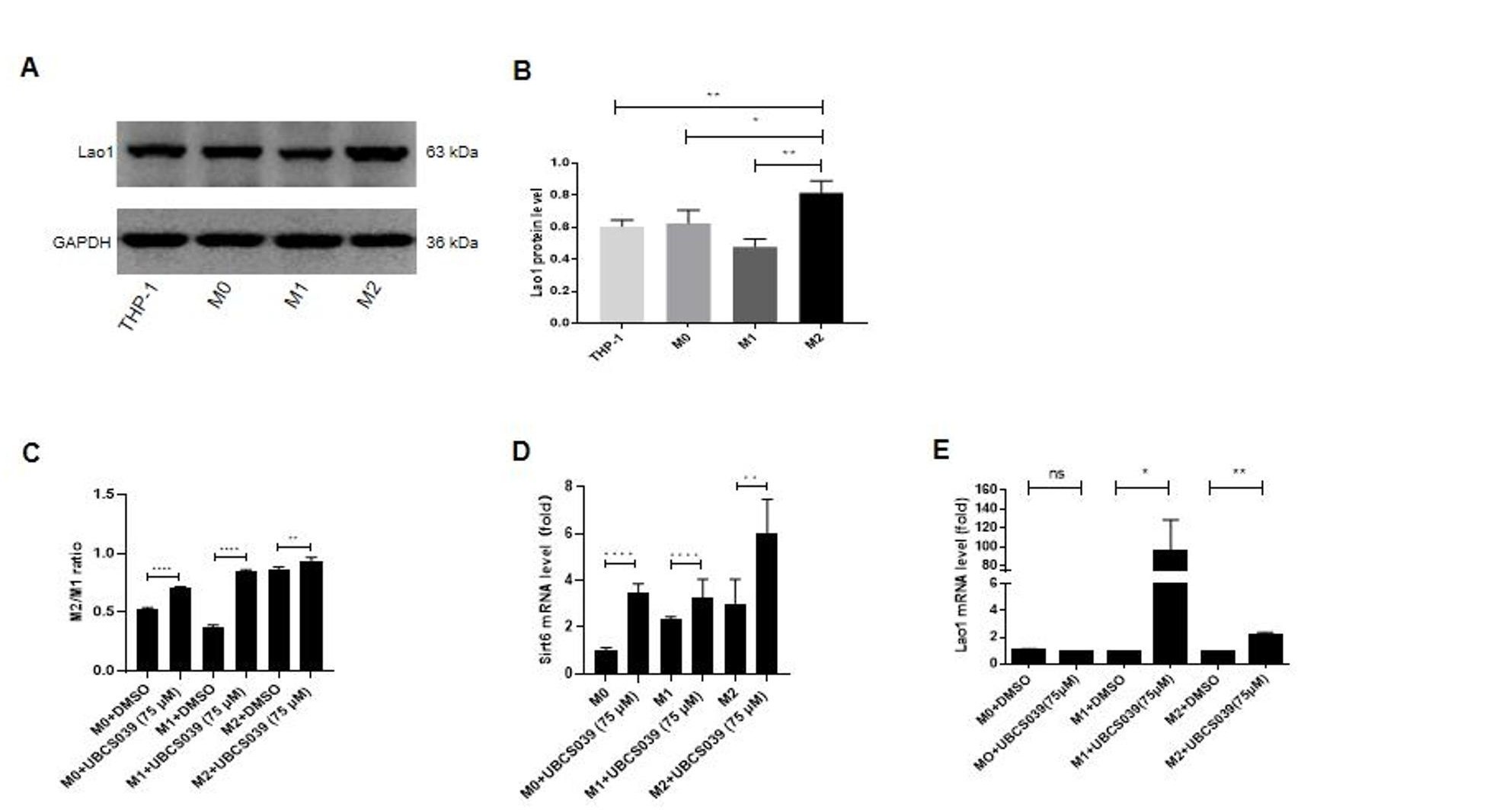
The effect of UBCS039 on macrophage polarization. M0, M1 and M2 macrophages were induced from THP-1 cells in vitro. (**A**) Lao1 and GAPDH expression levels were determined via western blot analysis in THP-1, M0, M1 and M2 macrophages. (**B)** Lao1 protein expression was normalized to that of GAPDH. Lao1 expression is increased in M2 macrophages. (**C)** M0-, M1- and M2-type macrophages were incubated with 75 µM UBCS039. Flow cytometry revealed an increased M2/M1 ratio in M0-, M1- and M2-type macrophages following UBCS039 treatment. (**D)** Sirt6 expression levels in macrophages were determined via real-time PCR. Following incubation with UBCS039, Sirt6 mRNA levels were significantly elevated in M0-, M1- and M2-type macrophages. (**E)** Lao1 expression was measured via real-time PCR. Following incubation with UBCS039, Lao1 mRNA levels were significantly elevated in the original M1- and M2-type macrophage groups, especially in the M1-type macrophage group. The least significant difference (LSD) test was used for comparisons between two groups. * indicates *P* < 0.05, ** indicates *P* < 0.01, *** indicates *P* < 0.001, and **** indicates *P* < 0.0001

We examined how UBCS039 affects macrophage polarization in vitro. THP-1 cells were cultured and induced to differentiate into M0-type macrophages, and M1- and M2-type macrophages were generated in vitro from M0 macrophages. UBCS03975 at a final concentration of 75 µM was added to the wells containing each type of macrophage. Figure 10A **and B** show that Lao 1 protein expression was particularly elevated in M2 macrophages compared with M1 macrophages, as determined by western blot analysis. The experiment was repeated 3 times. The whole western blot membrane and original data are shown in Supplementary File 7. When M0, M1 and M2 macrophages received 75 µM UBCS039 treatment, the M2/M1 macrophage ratio was elevated in the original M0- and M1-type macrophages, especially in the M1-type macrophage group, but had little effect on M2-type macrophages, as determined by flow cytometry (Fig. 10C and Supplementary File 8). These results indicate that UBCS039 treatment strongly stimulated M0- and M1-type macrophage polarization to M2-type macrophages in vitro. The high M2/M1 ratio of M0 macrophages indicates that more M0 macrophages than M1-type macrophages were polarized to M2-type macrophages following UBCS039 treatment. Moreover, Sirt6 mRNA expression also increased in M0-, M1- and M2-type macrophages following treatment with UBCS039, as determined by real-time PCR. Hence, UBCS039 increased Sirt6 protein expression during macrophage polarization to the M2 type (Fig. 10D). Moreover, real-time PCR revealed increased Lao1 expression in M1 and M2 macrophages, especially in the M1 subtype, following UBCS039 treatment compared with M0 macrophages (Fig. 10E), indicating that Lao1 expression is increased when M1 macrophages polarize into M2 macrophages.

## Discussion

To establish tumor-bearing mice for our current study, we subcutaneously injected different lines of human-derived tumor cells into BALB/c nude mice. Tumor growth curves, PET–CT and in vivo bioluminescence imaging all revealed larger tumor tissues in mice that were given UBCS039 than in control mice. We also detected increased Sirt6 expression in tumor tissues from mice after UBCS039 treatment compared with that in the control mice. These observations demonstrated that the upregulation of Sirt6 by UBCS039 accelerated tumor growth, which can explain the inverse link between poor patient survival and high Sirt6 expression in tumors, as reported in clinical studies [5].

To investigate how Sirt6 impacts immune surveillance in mice with tumor grafts, we measured the levels of immunoregulation-related cytokines, genes and other factors in the peripheral blood and the tumor tissues of these mice. GM-CSF, IL-1α, IL-10, IL-12p70, IL-17 and IL-23 concentrations in the serum were elevated in such mice injected with MB-231 cells following UBSC039 pretreatment, and IFN-γ levels were significantly decreased. UBCS039-pretreated tumor-bearing mice also presented increased CRP levels in their blood. Additionally, mice that were given UBCS039 presented elevated levels of ADO in their peripheral blood along with increased expression of PD-L1, NF-κB and PD-1 in their tumors. Tumor cells have been reported to produce high amounts of ADO [33]. As we described in the introduction, high levels of ADO and PD-1/PD-L1 suppress the immune response. Through the NF-kB cascade, IFN-γ efficiently induces cell death and has potent anticancer effects [34, 35]. The above results demonstrate that the upregulation of Sirt6 by UBCS039 treatment suppressed immune surveillance to favor tumor growth. Additionally, CRP is a type of protein whose levels rise sharply in plasma when the body is infected or injured. In the present study, CRP levels increased accordingly after the mice were grafted with human tumor cells, as their bodies had been invaded by foreign substances. Our real-time PCR and immunofluorescent assay results revealed a much greater proportion of M2-type macrophages in tumor tissues from UBCS039-pretreated mice than in those from DMSO-pretreated and PBS-pretreated mice. Additionally, flow cytometry and real-time PCR revealed that UBCS039 treatment increased the M2/M1 macrophage ratio in M0- and M1-type macrophages in vitro. Increased proportions of M2-type macrophages, also termed TAMs, promote tumor neoangiogenesis, cell proliferation, epithelial-to-mesenchymal transition, metastasis matrix remodeling and lymphangiogenesis [36]. The results of the present study suggest that the upregulation of Sirt6 induced by UBCS039 treatment increased the proportion of M2-type macrophages or TAMs in the TME to form an microenvironment conducive to tumor growth. Others also found that Sirt6 activation by UBCS039 can shift macrophages from M1 type to M2 type in sepsis-induced acute respiratory distress syndrome, hepatic injury and peripheral nerve injury [26, 31, 37]. As in the present study, we previously examined Sirt6 mRNA expression levels in several cancer cell lines (A2780, HeLa, Huh7, MBA-MD-231, SMMC-7721 and SW480) with UBCS039 treatment and compared the expression level with corresponding normal cells. We not only detected increased expression of Sirt6 following UBCS039 treatment in these cell lines but also found that the Sirt6 expression level varied among these tumor cell lines [29]. The measurement results are similar to those in the current study in tumor tissues. Because different tumor cell types show different sensitivities to UBCS039 treatment and the subsequent deacetylation activity of Sirt6, these tumor cell lines show different tumor growth levels after injection into mouse models. CD38 is a multifunctional extracellular enzyme located on the cell surface that exerts NADase and cyclase activities. CD38 is expressed in immune cells and tumor cells [38]. In our previous study, we found the inhibiting CD38 activity with anti-CD38 siRNA, C3G (a natural CD38 inhibitor), 78c (a chemical CD38 inhibitor) and anti-CD38 antibody increased Sirt6 expression in H9C2 cells and CD38 + NK cells and in D-gal-induced acute aging mice; moreover, inhibiting Sirt6 expression with anti-Sirt6 siRNA increased CD38 expression. CD38 and Sirt6 could downregulate each other to form a closed-loop regulation mechanism, but this suggestion is based on NK cells and H9C2 cardiomyocytes [30, 39, 40]. Sirt6 is a member of the NAD-dependent family of sirtuins. Other enzymes that regulate NAD + supply also likely affect Sirt6 expression and activity. The impact of NAD + depletion on the differentiation of mouse and human primary monocytes/macrophages was studied in vitro. Nicotinamide phosphoribosyltransferase (NAMPT) can reduce the expression and activity levels of NAD-dependent enzymes, including PARP1 (poly(ADP-ribose) polymerase 1), Sirt6 and CD38 [41]. Another study found that the ubiquitin ligase CHIP (carboxyl terminus of Hsp70-interacting protein) prevents Sirt6 degradation through noncanonical ubiquitination [42]. The Sirt6 deacetylase is a key regulator of mammalian genome stability, metabolism and lifespan [43]. To understand how Sirt6 is involved in tumorigenesis and immune surveillance, transcriptome analysis was performed on tumor tissues originating from MB-231 cells in UBCS039-pretreated mice. The analysis revealed a specific increase in Lao1 expression in the tumor tissues of UBCS039-pretreated mice but not in the tissues from DMSO-pretreated mice and PBS-pretreated mice. The results were confirmed by western blotting as well as real-time PCR in tumor tissues from MD-231, HeLa, Hcc827 and LM3 cell-injected mice. Lao1, also known as interleukin 4-induced 1 (IL4I1), has been reported to control M2 macrophage polarization. Lao1 overexpression induces bone marrow-derived macrophage differentiation to M2 type, whereas M1 type-related cytokines are inhibited [44]. Lao1 protein expression is upregulated in tumor cells and is considered a protective factor or prognostic biomarker for many cancer types. As a prognostic indicator, the Lao1 methylation level can be utilized [45]. Additionally, the level of TAM infiltration was positively correlated with the expression of Lao1 in pancancerous tissues [45]. Thus, Lao1 acts as a negative regulator of antitumor immunity [46, 47]. In our in vitro experiments, Lao1 expression increased in M1 macrophages after UBCS039 treatment, and M1-type macrophages were subsequently polarized toward the M2 type. We thus suggest that the increase in Sirt6 activity caused by UBCS039 augmented Lao1 expression in macrophages to promote their polarization to the M2 type to favor tumor growth. Thus far, there has been no report regarding the involvement of Lao1 in the regulation of Sirt6 expression. Others have reported that Lao1 in TAMs also suppresses T-cell responses, leading to immune system escape [48–50]. Tryptophan is converted to kynurenine and indole-3-pyruvic acid when IFN-γ activates indoleamine 2,3-dioxygenase 1 along with Lao1 [48]. After *Mycobacterium tuberculosis* infection, Sirt2 and Sirt5, the two members of the Sirt family, control these pathways [51]. Additionally, Lao1 controls macrophage polarization and prevents T-cell activation by reducing arginine as well as L-tryptophan and producing IL-10 [44]. L-Phenylalanine and L-arginine are the main products catabolized by the secreted L-amino acid oxidase protein Lao1 [52]. Interestingly, our KEGG enrichment analysis revealed activated tryptophan biosynthesis and tryptophan metabolism in tumor tissues from UBCS039-pretreated mice. We also detected a decrease in IFN-γ levels in tumor-bearing mice following UBCS039 treatment. These results suggest that Sirt6 may increase tumor growth by increasing Lao1 expression to elevate the number of TAMs in the TME and activate Lao1-controlled tryptophan metabolism. In our study, NK cells were increased in tumor-bearing nude mice pretreated with UBCS039. The innate immune system’s NK cells are needed for immune surveillance. An expansion of the NK cells was detected in thyroid cancer patients with poor overall survival [53]. In esophageal squamous cell carcinoma intratumoral tissues, CD49a + NK cells with high CD103 and CD69 expression were increased in the CD56bright NK cell subset [54]. NK cells mainly perform a killing function, while some NK cells function in immune regulation [55, 56]. According to our study, colorectal cancer patients have a much greater CD38 + NK cell proportion in the peripheral blood. The level of CD38 + NK cells was linked to poor prognosis as well as lymph node metastasis [57]. CD38 + NK cells from tumor patients can stimulate CD4 + T cells to differentiate into Tregs and macrophages to polarize into M2 type, which constitutes an immune microenvironment conducive to the growth of tumor cells. CD38 + NK cells from tumors exhibit decreased IFN-g production and increased NF-kB levels, and the NK cell subset al.so expresses high levels of ADO and PD-1 [58, 59], which is in agreement with the current results. One study reported that Sirt6 inhibits NK cells’ ability to fight tumors in murine inflammatory colorectal cancer [60]. Unfortunately, the present study did not further examine the subtypes of increased NK cells in tumor-bearing mice.

In the present study, we used UBCS039 at a concentration of 75 µM and incubated it with macrophages in an in vitro study. We also injected nude mice with UBCS039 at 10 mg/kg body weight. Other studies used similar concentrations to treat mouse models and cultured cells. They observed the changes in Sirt6 protein expression after treatment using western blot analysis [26, 31]. In our previous study, Sirt6 protein expression was significantly increased in H9c2 cells treated with UBCS039 at a concentration of 100 µM using western blot analysis [30]. Additionally, we observed the effect of UBCS039 treatment on macrophage polarization and tumor growth in tumor-bearing mice in the present study. Thus, the present study found that UBCS039 at a concentration of 75 µM can activate Sirt6 to exert its deacetylation activity.

Our current research has several limitations. Lao1 knockdown experiments should be performed to verify how Lao1 functions in M2 macrophage polarization during tumorigenesis, though others have confirmed the essential role of Lao1 in macrophage polarization [44]. Additionally, we did not detect changes in dendritic cells, splenetic cells or bone marrow cells in tumor-bearing mice that were given UBCS039. In the present study, we used nude mice (BALB/c FOXN1-/-) to explore the effect of Sirt6 on tumor growth and immune surveillance. The Foxn1 gene mutation leads to the development of thymus defects and T-cell deficiency. Because we used human tumor cell lines rather than mouse-derived tumor cells, we had to use nude mice to establish the tumor-bearing model, which is a routine method to investigate the effect of anti-tumor drugs. Because nude mice do not produce T cells, our investigation of the effect of Sirt6 on immune surveillance is not complete and could even be considered one-sided. Therefore, we will perform similar studies with humanized mice or immunocompromised mice injected with homogeneous tumor cells to confirm the destructive effects of UBCS039 and Sirt6 activity on immune surveillance. Additionally, we had incubated the human HeLa, A2780, MBA-MD-231, Huh7, SW480, and SMMC-7721 cell lines with UBCS039. After removing residual UBCS039 from the cells, we cultivated these tumor cells in a transwell system with human naive CD4 + T cells to monitor T-cell differentiation. After being cultured with UBSC039-pretreated tumor cells, the percentage of Tregs among CD4 + T cells was elevated significantly. In the tumor cells that were given UBS039, the PD-L1 and Sirt6 expression levels were also elevated, as was PD-1 expression in cocultured CD4 + T cells. Moreover, in the coculture medium, ADO levels increased, but IFN-α2, IL-10, monocyte chemoattractant protein-1 (MCP-1), and IFN-γ levels decreased [29]. The in vitro experimental results with CD4 + T cells partially compensate for the deficiency of T cells when exploring immune surveillance in nude mice. It is possible that, under the condition of a complete immune system, increased Sirt6 activity and expression in tumors caused by UBCS039 treatment promoted both the differentiation of CD4 + T cells to Tregs and macrophage polarization to M2-type macrophages to suppress immune surveillance. It has been reported that Lao1 also increases Treg differentiation [52] and that Tregs produce high amounts of ADO [61]. We are conducting step-by-step investigations into how Sirt6 affects the immune microenvironment. In the initial step, we used immunodeficient nude mice to determine the effect of UBCS039 on tumor growth and to define the optimal experimental conditions. The current study focuses on the results of this initial step.

## Conclusions

Increased Sirt6 expression and activity induced by UBCS039 can accelerate tumor growth by promoting macrophage polarization to the M2 type or TAMs through the upregulation of Lao1 expression, which can suppress immune surveillance of tumor cells. This finding provides a possible mechanism for high TAM numbers in tumor tissues. Sirt6 upregulation also activates tumor cells to augment PD-L1, ADO, NF-κB, and PD-1 expression levels and lower IFN-g gamma production. Additionally, UBCS039 changes the immune regulation by NK cells to disturb immune surveillance. The above results explain for the first time the poor survival of patients with high Sirt6 expression. Sirt6 may promote tumor growth and is a potential target for tumor therapy. The inhibition of Sirt6 activity and expression may exert antitumor effects in the clinic.

## Supplementary Information

Supplementary Material 1

Supplementary Material 2

Supplementary Material 3

Supplementary Material 4

Supplementary Material 5

Supplementary Material 6

Supplementary Material 7

Supplementary Material 8

## Data Availability

The datasets used during the study are available from the corresponding author on reasonable request.
