# Supplementary material for: Sirt6 promotes tumor growth and suppresses immune surveillance
Source: Cancer Cell Int. 2026 Jan 13;26:20. doi: 10.1186/s12935-025-04125-x (PMC12809971; doi:10.1186/s12935-025-04125-x)
Supplement: Supplementary file 1 — Supplementary Material 1 [file 12935_2025_4125_MOESM1_ESM.docx]

**Supplementary file 1. Primer sequences for real-time PCR**

**Arg1**

Forward: 5’ AGGCGCTGTCATCGATTTCT 3’

Reverse: 5’ TGGAGTCCAGCAGACTCAAT 3’

**CD206**

Forward: 5’ CTCTGTTCAGCTATTGGACGC 3’

Reverse: 5’ CGGAATTTCTGGGATTCAGCTTC 3’

**GAPDH**

Forward: 5’ CTTCATTGACCTCAACTACATGGTCTA 3’

Reverse: 5’ GATGACAAGCTTCCC ATTCTCAG 3’

**iNOS**

Forward: 5’ GCGCTCTAGTGAAGCAAAGC 3’

Reverse: 5’ AGTGAAATCCGATGTGGCCT 3’

**Lao1**

Forward: 5’ CCCTCCCGCTTCATTTACTATCCC 3’

Reverse: 5’ GCAAGGTCATCCAGCACAACATC 3’

**NF-kB**

Forward: 5’ ATGGGAAACCGTATGAGCCTGTG 3’

Reverse: 5’ AGTTGTAGCCTCGTGTCTTCTGTC 3’

**PD-1**

Forward: 5’ TGTTGACAGCAGGGAAGGAAAGG 3’

Reverse: 5’ CTAGGTGTGAAGGAGAGCCAGAAC 3’

**PD-L1**

Forward: 5’ TGAGCAAGTGATTCAGTTTGTG 3’

Reverse: 5’ CATTTCCCTTCAAAAGCTGGTC 3’

**Sirt6**

Forward: 5’ GGCTACGTGGATGAGGTGAT 3’

Reverse: 5’ GGCTCAGCCTTGAGTGCTAC 3’

**TNF-α**

Forward: 5’ CTCAGCGAGGACAGCAAGG 3’

Reverse: 5’ AGGGACAGAACCTGCCTGG 3’
