# Supplementary figures and images for: Sirt6 promotes tumor growth and suppresses immune surveillance
Source: Cancer Cell Int. 2026 Jan 13;26:20. doi: 10.1186/s12935-025-04125-x (PMC12809971; doi:10.1186/s12935-025-04125-x)

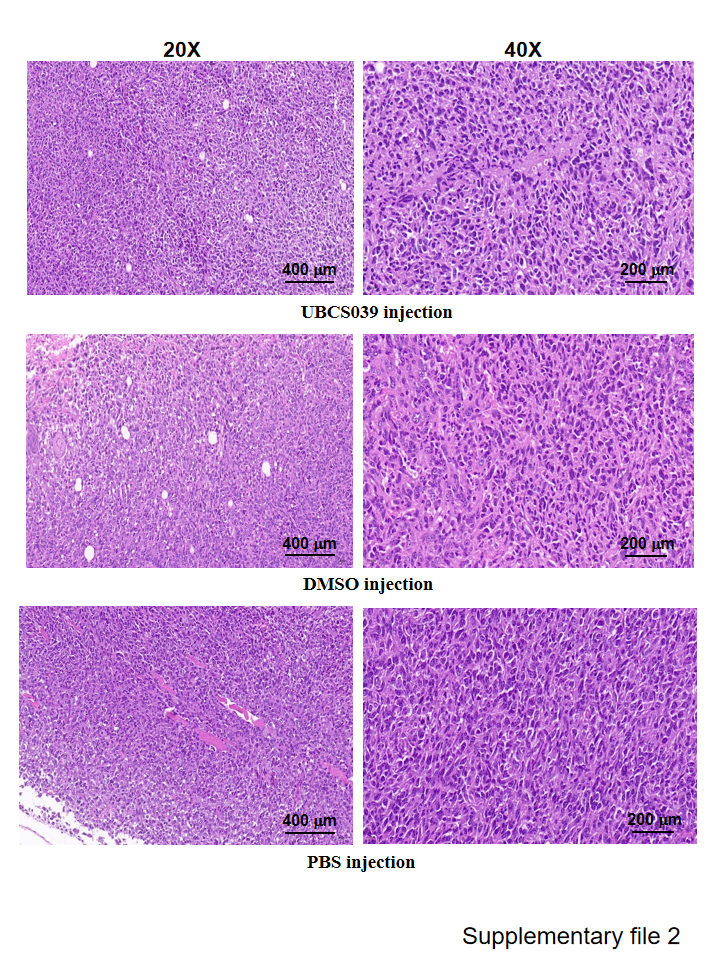

Supplement: Supplementary file 2 — Supplementary Material 2 [file 12935_2025_4125_MOESM2_ESM.tif]

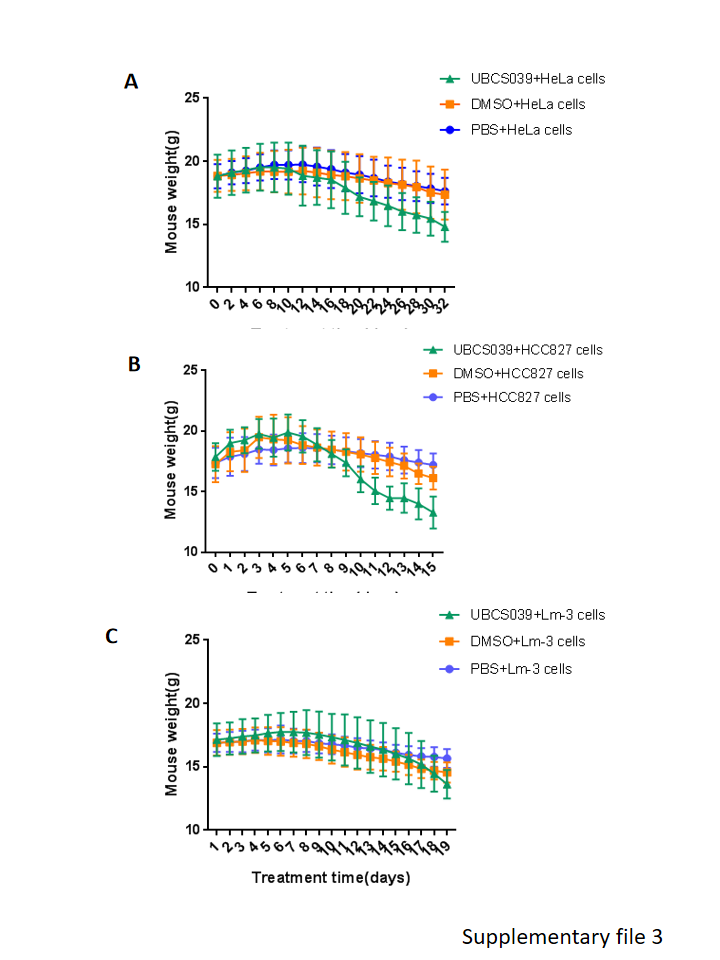

Supplement: Supplementary file 3 — Supplementary Material 3 [file 12935_2025_4125_MOESM3_ESM.tif]

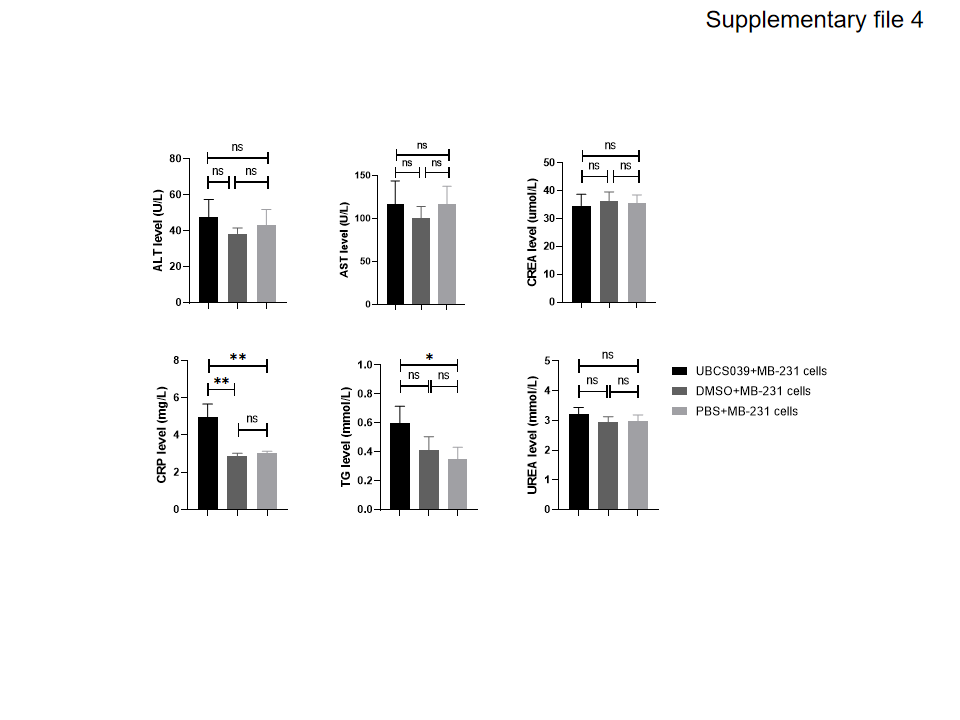

Supplement: Supplementary file 4 — Supplementary Material 4 [file 12935_2025_4125_MOESM4_ESM.tif]

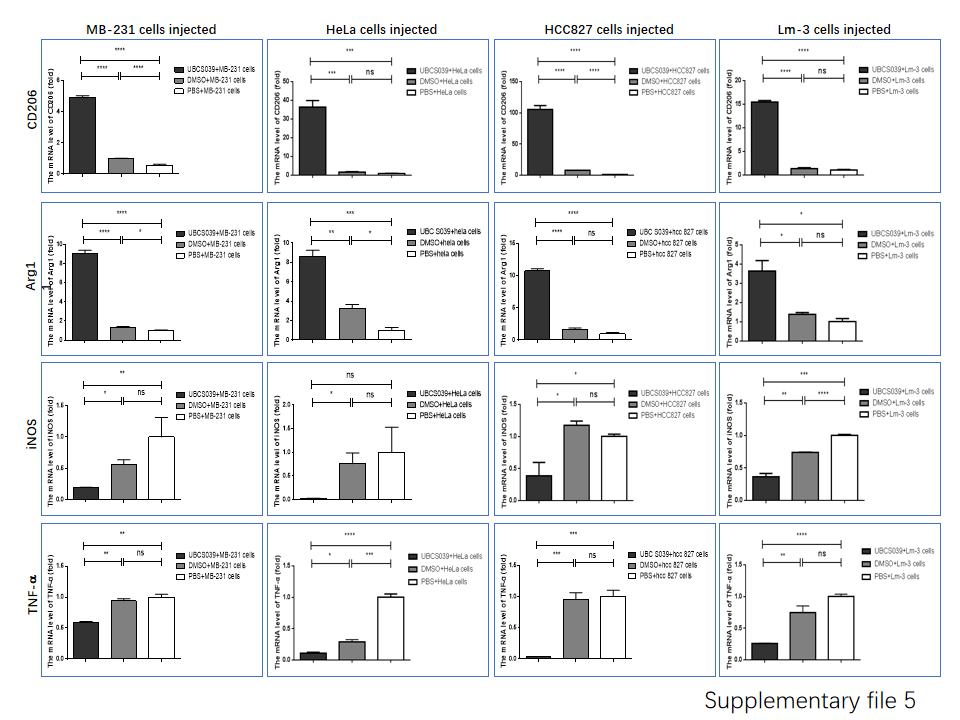

Supplement: Supplementary file 5 — Supplementary Material 5 [file 12935_2025_4125_MOESM5_ESM.tif]

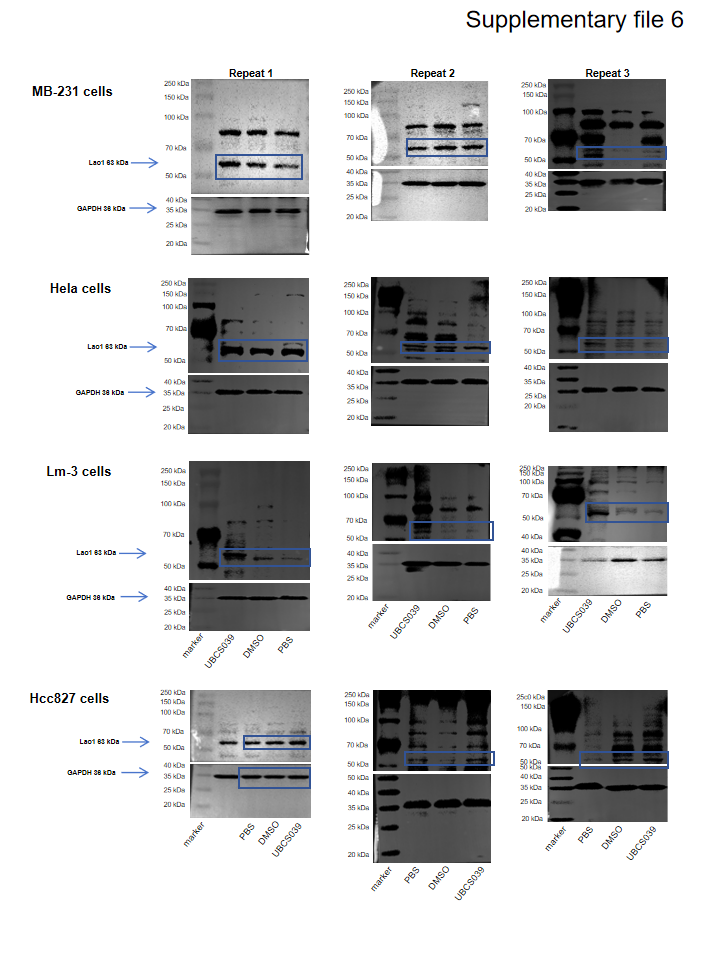

Supplement: Supplementary file 6 — Supplementary Material 6 [file 12935_2025_4125_MOESM6_ESM.tif]

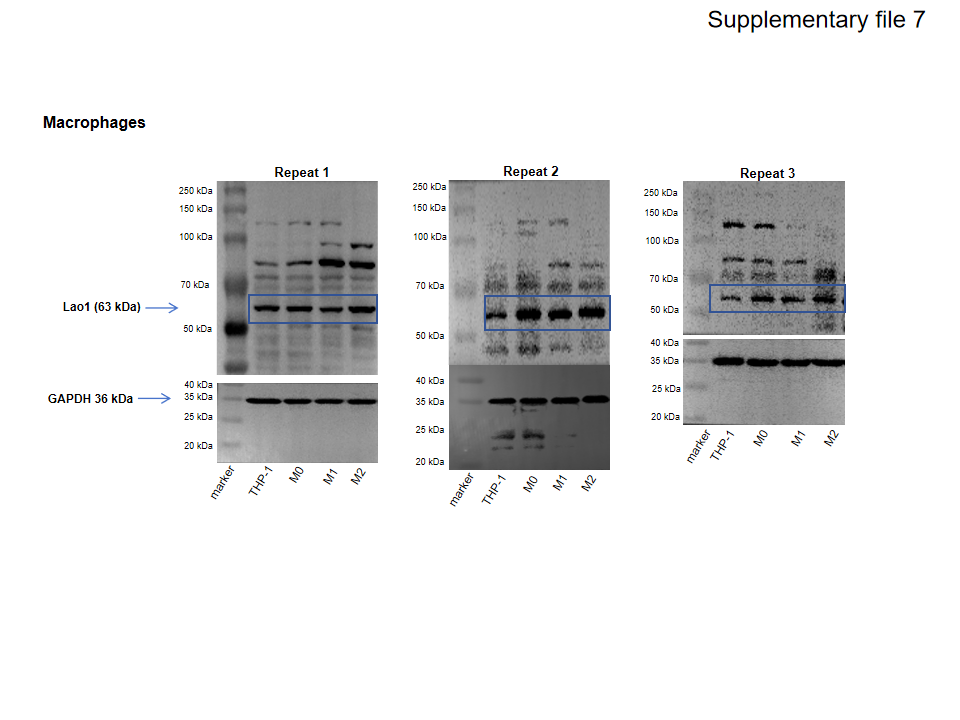

Supplement: Supplementary file 7 — Supplementary Material 7 [file 12935_2025_4125_MOESM7_ESM.tif]

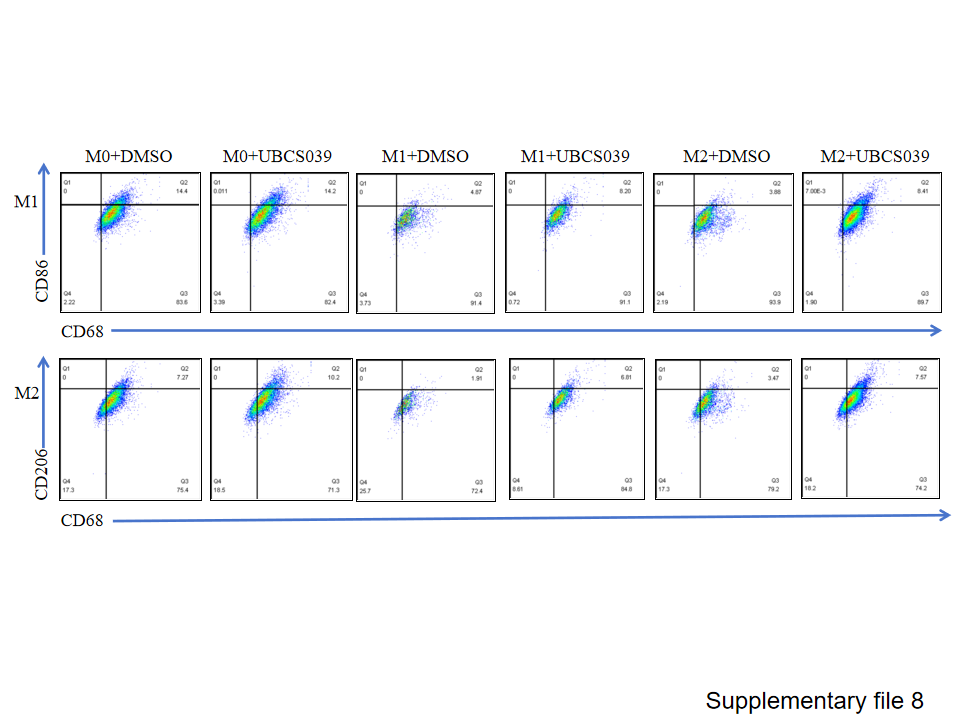

Supplement: Supplementary file 8 — Supplementary Material 8 [file 12935_2025_4125_MOESM8_ESM.tif]
